# Development of a Novel Avian Vaccine Vector Derived From the Emerging Fowl Adenovirus 4

**DOI:** 10.3389/fmicb.2021.780978

**Published:** 2021-12-01

**Authors:** Qing Pan, Yu Zhang, Aijing Liu, Hongyu Cui, Yulong Gao, Xiaole Qi, Changjun Liu, Yanping Zhang, Kai Li, Li Gao, Xiaomei Wang

**Affiliations:** ^1^State Key Laboratory of Veterinary Biotechnology, Harbin Veterinary Research Institute, Chinese Academy of Agricultural Sciences, Harbin, China; ^2^Jiangsu Co-innovation Centre for Prevention and Control of Important Animal Infectious Diseases and Zoonoses, Yangzhou University, Yangzhou, China

**Keywords:** FAdV-4, vaccine vector, HHS, non-essential regions, IBDV

## Abstract

Severe hepatitis-hydropericardium syndrome (HHS) associated with a novel viral genotype, fowl adenovirus 4 (FAdV-4), has emerged and widely spread in China since 2015, causing severe economic losses to the poultry industry. We previously reported that the hexon gene is responsible for pathogenicity and obtained a non-pathogenic hexon-replacement rHN20 strain; however, the lack of information about the non-essential regions for virus replication limits the development of a FAdV-4 vector. This study first established an enhanced green fluorescent protein (EGFP)-indicator virus based on the FAdV-4 reverse genetic technique, effective for batch operations in the virus genome. Based on this, 10 open reading frames (ORFs) at the left end and 13 ORFs at the right end of the novel FAdV-4 genome were deleted separately and identified as non-essential genes for viral replication, providing preliminary insertion sites for foreign genes. To further improve its feasibility as a vaccine vector, seven combinations of ORFs were successfully replaced with EGFP without affecting the immunogenicity of the vector backbone. Finally, a recombinant rHN20-vvIBDV-VP2 strain, expressing the VP2 protein of very virulent infectious bursa disease virus (vvIBDV), was rescued and showed complete protection against FAdV-4 and vvIBDV. Thus, the novel FAdV-4 vector could provide sufficient protection for HHS and efficient exogenous gene delivery. Overall, our findings systemically identified 23 non-essential ORFs for FAdV-4 replication and seven foreign gene insertion regions, providing valuable information for an in-depth understanding of the novel FAdV-4 pathogenesis and development of multivalent vaccines.

## Introduction

Adenoviruses (family *Adenoviridae*) are non-enveloped, double-stranded DNA viruses that infect multiple vertebrates, including mammals, birds, fish, and reptiles (Greber, 2020). According to the International Committee on Taxonomy of Viruses (ICTV), the family *Adenoviridae* is classified into five genera: *Adenovirus, Aviadenovirus, Chadenovirus, Mastadenovirus*, and *Siadenovirus.* Fowl adenoviruses (FAdVs) belong to the genus *Aviadenovirus* and are defined as 5 species (A–E) and 12 serotypes (1–7, 8a–8b, 9–11) (Steer et al., 2009). Pathogenic FAdV strains infect chickens, causing inclusion body hepatitis (IBH), hepatitis-hydropericardium syndrome (HHS), and gizzard erosion (GE) (Liu et al., 2016; Li et al., 2018; Schachner et al., 2018; Wang et al., 2020). A severe outbreak of HHS by the novel FAdV-4 has occurred in China since 2015, causing severe economic losses to the poultry industry (Liu et al., 2016; Niu et al., 2016; Xia et al., 2017; Li et al., 2018).

With the development of gene editing and reverse genetic technology, various viruses have been successfully modified as viral vectors, such as poxvirus (Lévy et al., 2020), herpes virus (Herold et al., 2002), Newcastle disease virus (NDV) (Sun et al., 2020), and adenovirus (Wu et al., 2021). For instance, Fowlpox virus was used as a recombinant vaccine vector against mammalian and poultry viruses (Taylor et al., 1988; Boyle and Heine, 1993); Gallid herpesvirus 2 (GaHV-2), expressing the infectious bursal disease virus (IBDV) VP2 protein, was used for the prevention and control of Marek’s disease virus (MDV) and IBDV (Li et al., 2016); whereas NDV was reported to be immunogenic and efficacious against avian influenza HA (Zhang X. et al., 2021). In addition, human adenovirus vectors have been widely used for gene therapy (Arnone et al., 2021) and vaccines against emergent viruses such as Ebola (Li et al., 2017) and SARS-Cov-2 (Tostanoski et al., 2021), however, non-human and non-mammalian adenoviruses, including FAdVs, are used less frequently as vectors.

The genome of human and mammalian adenoviruses (*Mastadenovirus*) is approximately 34–38 kb, including early transcription genes (E1, E2, E3, and E4) and late transcription genes (L1, L2, L3, L4, and L5). The genes located in E1 and E3 were identified as essential and non-essential genes, respectively, providing a theoretical basis for developing adenovirus vectors (Tatsis and Ertl, 2004). Nevertheless, FAdVs have the longest genome (43–36 kb) among adenoviruses without E1, E3, or E4 regions, whereas only the E2 and L1–L4 regions in the central part of the genome are conserved with mammalian adenoviruses. Previous studies on the chicken embryo lethal orphan (CELO) strain (non-pathogenic FAdV-1) showed that 16 of the 22 unassigned open reading frames (ORFs) are non-essential for virus replication and that the rightmost segment of the genome (containing ORF9, ORF10, and ORF11) can be replaced by an enhanced green fluorescent protein (EGFP) expression cassette (Michou et al., 1999; François et al., 2001). Nevertheless, the CELO-vectored vaccine could not protect chickens against HHS, and thus, a novel FAdV-4-based vaccine vector is urgently needed.

The inactivated FAdV-4 oil-emulsified vaccine can induce full protection (Pan et al., 2017b), whereas the live-vectored bivalent or multiple vaccines can further reduce the production cost and workload. Given the emergence, high pathogenicity, and lack of knowledge of the non-essential regions of the novel FAdV-4, limited progress has been achieved in vector development. We previously reported that the hexon gene played a critical role in FAdV-4 pathogenicity and obtained a non-pathogenic rHN20 strain (Zhang Y. et al., 2021). In this study, 10 ORFs at the left end and 13 ORFs at the right end of the novel FAdV-4 genome were separately deleted and identified as non-essential regions for virus replication based on an EGFP-indicator virus. Furthermore, seven combinations of ORFs were successfully replaced with EGFP without affecting the immunogenicity of the vector backbone. Finally, the FAdV-4 vector was used to deliver the VP2 protein of vvIBDV and provided complete protection against FAdV-4 and vvIBDV.

## Materials and Methods

### Animals and Ethics Statement

Specific pathogen-free (SFP) chickens obtained from Harbin Veterinary Institute of Chinese Academy of Agricultural Sciences (HVRI, CAAS) were housed in a negative-pressure environment at the Experimental Animal Center of HVRI. The birds had *ad libitum* access to water and feed. The study was approved by the Ethical and Animal Welfare Committee of HVRI (approval No. HVRI-IACUC-2021-0426-5).

### Virus, Cell, and Antibody

The highly pathogenic FAdV-4 strain HLJFAd15 (GenBank accession number KU991797) was isolated in our laboratory (Pan et al., 2017a), and the non-pathogenic strain rHN20 was constructed as described previously (Zhang Y. et al., 2021). All FAdV-4 viruses propagated in chicken hepatocellular carcinoma (LMH) cells. The vvIBDV HLJ0504 strain was also isolated in our laboratory, and the cell-adapted IBDV rGtHLJVP2 strain, inducing cytopathic effects (CPE) in a continuous cell line of chicken embryo fibroblasts (DF-1), was constructed as described previously (Gao et al., 2011). LMH cells were cultured in DMEM/F12 (Sigma-Aldrich, St. Louis, MO, United States), whereas DF-1 in DMEM (Sigma-Aldrich). Both the mediums were supplemented with 10% fetal bovine serum (Sigma-Aldrich), 100 μ ml^–1^ streptomycin, and 100 IU ml^–1^ penicillin at 37°C and 5% CO_2_. The anti-IBDV-VP2 monoclonal antibody was prepared in our laboratory.

### Construction of Recombinant Viruses

The FAdV-4 rHN20 fosmid was generated from the HLJFAd15 strain. Recombinant fosmids were engineered from rHN20 in *Escherichia coli* DH10B cells using the Counter-Selection BAC Modification kit (Gene Bridges, Heidelberg, Germany) as described previously (Zhang Y. et al., 2021). In brief, the rpsL-neo selection antibiotic cassette was inserted into the genome-expected sites, and rpsL-neo was replaced by a PCR-amplified product or single-stranded oligonucleotide with short homology arms.

The EGFP expression cassette from the pEGFP-N1 plasmid (Clontech, Mountain View, CA, United States) was inserted into the natural 1,966-bp deletion site (Pan et al., 2018) of rHN20 to construct the rHN20-EGFP recombinant fosmid. Next, each ORF was replaced with a single-stranded oligonucleotide to construct fosmids that individually deleted the 23 ORFs in the left and right ends of the FAdV-4 genome. Finally, the adjacent ORFs in the left and right ends of the FAdV-4 genome were divided factitiously into the L1, L2, L3, R1, R2, R3, and R4 regions. Every region was first replaced with EGFP to construct fosmids, in which EGFP replaced several adjacent ORFs as described above.

VP2 from the vvIBDV strain HLJ0504 cDNA was cloned into the pEGFP-N1 plasmid, and the EGFP ORF was replaced to form the VP2-expressing cassette. The VP2 cassette was inserted into the natural1966-bp deletion site of rHN20 to obtain the rHN20-vvIBDV-VP2 fosmid.

All recombinant fosmids were transfected into LMH cells after digestion with *Fse*I (Thermo-Fisher Scientific, Waltham, MA, United States) for 120 h post-transfection. Cells transfected with fosmids should be frozen and thawed three times, and the cell supernatant was inoculated into fresh LMH cells. The cell supernatant was collected for amplification and identification when apparent cytopathic effect (CPE) and/or green fluorescence appeared. The accuracy of all the recombinant viruses was confirmed using PCR. The VP2 expression of rHN20-vvIBDV-VP2 was identified using an indirect fluorescence antibody (IFA) assay with the monoclonal anti-IBDV-VP2.

### Virus Growth Curve

FAdV-4 was inoculated into LMH cells at a multiplicity of infection (MOI) of 0.01. The viruses were harvested at 12, 24, 48, 72, 96, and 120 h post-infection for plaque assay to determine viral titers.

### Immunization and Challenge Test

For rHN20 recombinant viruses expressing EGFP, 2-week-old SPF chickens were randomized into five groups (n = 10): a healthy control, a challenge control, and three groups, each immunized with 200 μl of rHN20, rDL3-EGFP, or r HN20-EGFP. At 14 d post-immunization, the immunized and challenge control groups were challenged with 2,000 plaque-forming units (PFU) rWT-FAdV4, whereas the healthy control group was administered DMEM/F12. Data on the morbidity and mortality of chickens were collected daily for 14 days. Viral loads were determined by collecting heart, liver, spleen, lung, kidney, thymus, and bursa from deceased or euthanized chickens at the test endpoint. The liver and thymus were used for histopathological analysis.

In addition, 50 two-week-old SPF chickens were randomized into five groups (*n* = 10): 2 groups were intramuscularly immunized with rHN20-vvIBDV-VP2, and the other 3 groups remained unimmunized. Neutralizing antibody titers in sera were determined at 7, 14, and 21 days post-immunization. At 21 days post-immunization, 10 immunized and 10 unimmunized chickens were challenged intramuscularly with 2,000 PFU virulent FAdV-4; the other 10 immunized and 10 unimmunized chickens were inoculated intranasally with 10^5^ EID_50_ vvIBDV. The left 10 chickens were used as healthy control. The chickens were monitored daily, euthanized, and necropsied at 7 days post-challenge.

### Viral Load Detection

Total DNA from different tissues was extracted using the AxyPrep Body Fluid Viral DNA/RNA Miniprep kit (Corning, Corning, NY, United States). Virus genome copies and cellular copies were quantified using Premix Ex Taq (TaKaRa, Kusatsu, Shiga, Japan) as described previously (Baigent et al., 2005; Pan et al., 2017c). Real-time PCR was performed using QuantStudio5 (Applied Biosystems, Waltham, MA, United States). Viral loads were expressed as the number of viral genome copies per 10^6^ cells (copies 10^–6^ cells) (Zhang Y. et al., 2021).

### Histopathological Analysis

The tissues were fixed in 10% formalin and embedded in paraffin. The sections were stained with hematoxylin and eosin (H&E) and observed under a microscope.

### Virus-Neutralization Assay

After inactivation at 56°C for 30 min, each serum sample was filtered through a 0.22-μm pore size filter. A twofold serial dilution of the serum was mixed with 200 median tissue culture infectious dose (TCID_50_) of the FAdV-4 rWT-EGFP strain or IBDV rGtHLJVP2 strain in a 96-well plate, incubated at 42°C for 1 h, and then added to the LMH or DF1 cells. After being cultured at 37°C for 72 h, IBDV CPEs were observed. After being cultured at 37°C for 6 day, the GFP expressed in FAdV-4 was visualized by fluorescence microscopy.

### Statistical Analysis

Two-way ANOVA with Tukey’s multiple comparison test was used for identifying significant differences. Significance was set at *p* < 0.05. Statistical analysis was performed with GraphPad (GraphPad Software, San Diego, CA, United States).

## Results

### Construction of rHN20-Enhanced Green Fluorescent Reporter Virus

**FIGURE 1:**
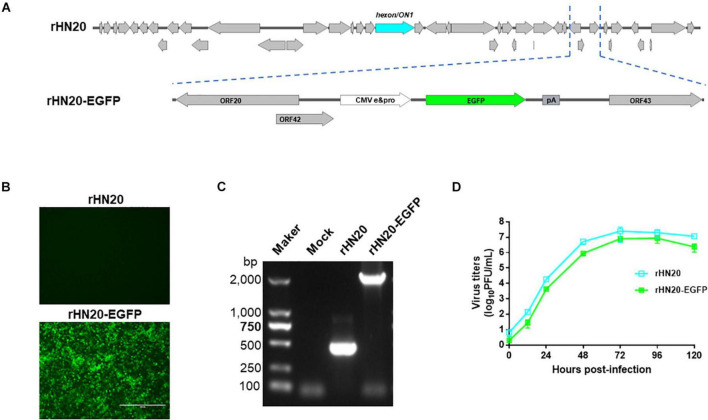
Construction of the rHN20-EGFP recombinant virus. **(A)** Diagram of rHN20-EGFP. **(B)** Green fluorescence in LMH cells infected with rHN20 and rHN20-EGFP. **(C)** PCR identification of rHN20-EGFP. **(D)** Replication kinetics of rHN20 and rHN20-EGFP.

To facilitate visualization of the virus on cells, we inserted an enhanced green fluorescent (EGFP) expression cassette on the natural 1,966 bp deletion between ORF42 and ORF43 of the FAdV-4 rHN20 strain (Figure 1A). The results showed that the rescued recombinant virus rHN20-EGFP strain could produce green fluorescence in LMH cells, whereas rHN20 could not (Figure 1B). PCR amplification of rHN20 and rHN20-EGFP genomic DNA revealed a 400-bp band for rHN20 and an approximately 2,000-bp band for rHN20-EGFP (Figure 1C). Comparing the replicative capacity of rHN20-EGFP with that of rHN20 on LMH cells, we found that rWT-EGFP replicated efficiently, despite its slightly reduced replicative capacity (Figure 1D). Thus, we successfully obtained an EGFP-expressing FAdV-4 reporter virus.

### Identification and Characterization of Open Reading Frames in Both Terminal Regions of FAdV-4 Genome

**FIGURE 2:**
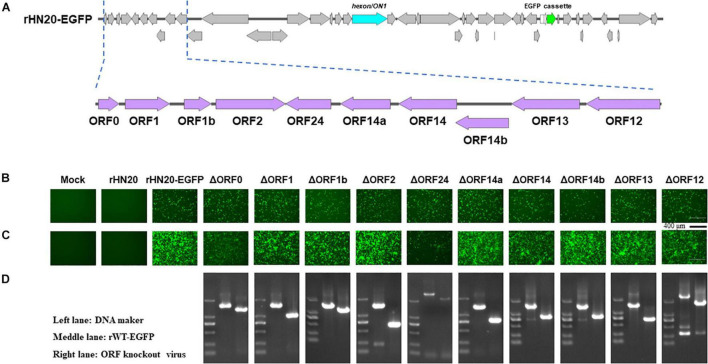
Identification of non-essential open reading frames (ORFs) in the left of the FAdV-4 genome. **(A)** Diagram of the genomic structure of rHN20-EGFP. Focal amplifications in purple represent ORFs unique to fowl adenoviruses on the left. **(B)** Green fluorescence in LMH cells transfected with linearized ORF knockout recombinant fosmids at 48 h post-transfection. Scale bar, 200 μm. **(C)** Green fluorescence in LMH cells infected with ORF knockout recombinant viruses at 72 h post-infection. Scale bar, 200 μm. **(D)** PCR identification of ORF knockout recombinant virus. A fragment of each recombinant virus containing the knockout ORF was amplified, and rWT-EGFP was used as a control.

**FIGURE 3:**
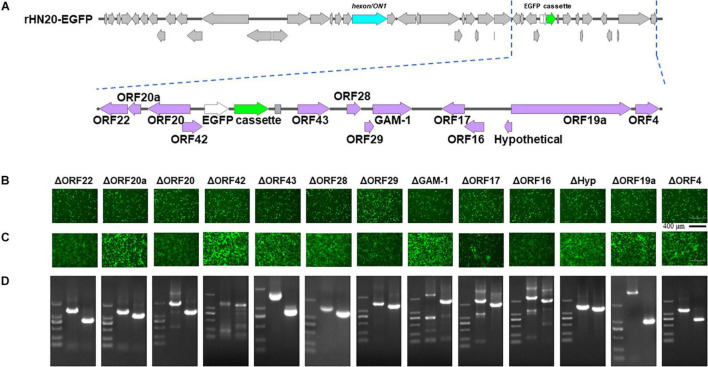
Identification of non-essential open reading frames (ORFs) in the right of the FAdV-4 genome. **(A)** Diagram of the genomic structure of rHN20-EGFP. Focal amplifications in purple represent ORFs unique to fowl adenoviruses on the right. **(B)** Green fluorescence in LMH cells transfected with linearized ORF knockout recombinant fosmids at 48 h post-transfection. Scale bar, 200 μm. **(C)** Green fluorescence in LMH cells infected with ORF knockout recombinant viruses at 72 h post-infection. Scale bar, 200 μm. **(D)** PCR identification of ORF knockout recombinant virus. A fragment of each recombinant virus containing the knockout ORF flanked was amplified, and rWT-EGFP was used as a control.

The ORFs at the left -and right-terminal regions of the FAdV genome are unique and not conserved within the *Adenoviridae* family (Davison et al., 2003). The FAdV-4 genome had 10 ORFs at the left end and 13 ORFs at the right end (Figures 2A, 3A). To determine whether these 23 ORFs are essential genes, they were individually deleted in the rHN20-EGFP fosmid, linearized, and transfected into LMH cells to recure ORF-knockout viruses. EGFP served as a reporter gene to monitor transfection success and viral production. At 48 h post-transfection, cells transfected with all fosmids engineered from rHN20-EGFP exhibited apparent green fluorescence, whereas mock and rWT-FAdV4 control cells showed no fluorescence (Figures 2B, 3B). Cell supernatants were harvested to infect new LMH cells, and all ORF-knockout recombinant viruses were successfully rescued (Figures 2C, 3C). Furthermore, all ORF knockout viruses were identified by PCR amplifying the knockout site fragment, resulting in a smaller band than the control (Figures 2D, 3D). Our data demonstrated that FAdV-4 could replicate after individual deletion of all 23 ORFs in both terminal regions of the viral genome.

### Construction of Recombinant Viruses With Enhanced Green Fluorescent Protein Substitutions of Several Open Reading Frames

**FIGURE 4:**
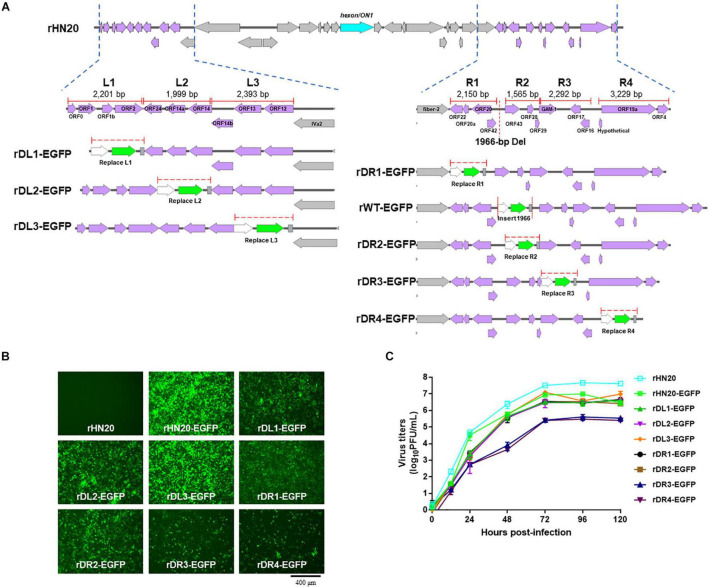
FAdV-4 non-essential genes serve as insertion sites of foreign gene. **(A)** Construction of recombinant FAdV-4 expressing EGFP gene. The left ORFs of FAdV-4 are divided into the L1, L2, and L3 regions, and the right ORFs are divided into the R1, R2, R3, and R4 regions. The length of each region is indicated and each region is individually replaced by an EGFP expression cassette. **(B)** Green fluorescence in LMH cells infected with different recombinant fluorescent viruses. **(C)** Replication kinetics of different recombinant fluorescent viruses.

To further confirm that the 23 ORFs in both terminal regions of the FAdV-4 genome were not essential for virus replication, the same ORFs in rHN20 were divided factitiously into L1, L2, L3, R1, R2, R3, and R4 regions that were each replaced by the EGFP cassette (Figure 4A). Seven recombinant viruses were generated, designated as rDL1-EGFP, rDL2-EGFP, rDL3-EGFP, rDR1-EGFP, rDR2-EGFP, rDR2-EGFP, and rDR3-EGFP. LMH cells infected with each of the seven recombinant viruses or rHN20-EGFP expressed green fluorescence (Figure 4B). All the eight EGFP-expressing recombinant viruses replicated efficiently in LMH cells (Figure 4C).

### Evaluation of Protective Efficacy of Live Recombinant Viruses Expressing Enhanced Green Fluorescent Protein

**FIGURE 5:**
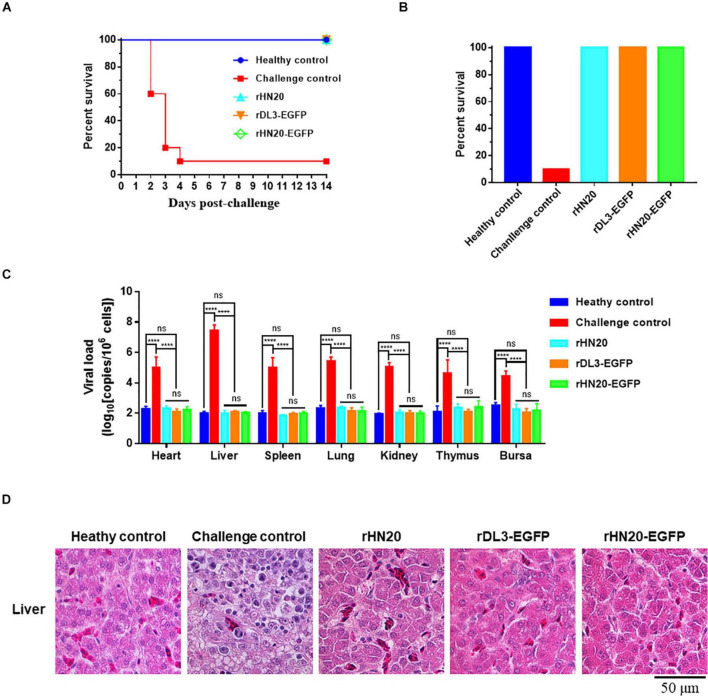
Protection against FAdV-4 lethal challenge by live recombinant fluorescent viruses. Two-week-old SPF chickens (*n* = 10) were injected with live rHN20, rDL3-EGFP, or rWT-EGFP and challenged with a lethal dose of FAdV-4 at 14 days post-injection. The healthy control was not treated. The challenge control was challenged but not immunized. **(A)** Survival curves of challenged chickens. **(B)** Survival rates of challenged chickens. **(C)** Viral loads in different tissues of challenged chickens. Heart, liver, spleen, lung, kidney, thymus, and bursa from dead or euthanized chickens were collected, and viral loads were determined using real-time PCR (*n* = 3). ****Significance at *P* < 0.0001; ns, no significance. **(D)** Histological examination of liver from challenged chickens. Scale bar, 50 μm.

An immunization and challenge test was performed to evaluate whether the recombinant FAdV-4 viruses expressing EGFP could be used as live vaccines. Two-week-old SPF chickens were immunized with live rHN20, rDL3-EGFP, or rHN20-EGFP. At 14 d post-immunization, the chickens were challenged with a lethal dose of FAdV4 HLJFAd15. As described in Figure 5A, all live recombinant FAdV-4 showed 100% protection. No clinical signs or death were observed in chickens pre-inoculated with live rHN20, rDL3-EGFP, or rHN20-EGFP within 14 d post-challenge, whereas the mortality of challenge control chickens reached 80% at 4 d post-challenge (Figure 5B). Moreover, high viral loads were observed in the tissues, especially livers, of the challenge control group at 14 day post-challenge, whereas almost no viral load was detected in the tissues of the immunized groups (Figure 5C). In line with these findings, massive pathological lesions and vacuolar necrosis were observed in the liver and thymus of the challenge control group, whereas no apparent pathological changes were found in any of the rHN20-, rDL3-EGFP, or rHN20-EGFP immunized groups (Figure 5D). Therefore, the insertion of EGFP did not impair the immunogenicity of FAdV-4.

### Construction of rHN20-vvIBDV-VP2 Recombinant Virus

**FIGURE 6:**
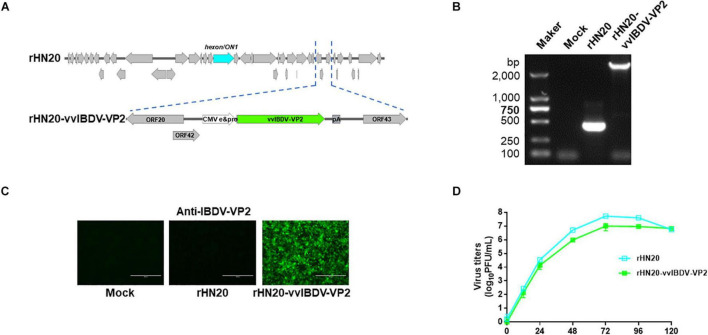
Construction of rHN20-vvIBDV-VP2 recombinant virus. **(A)** Diagram of rHN20-vvIBDV-VP2. **(B)** PCR identification of rHN20-vvIBDV-VP2. **(C)** Indirect immunofluorescence (IFA) identification of rHN20-vvIBDV-VP2 with monoclonal anti-IBDV-VP2. **(D)** Replication kinetics of rHN20 and rHN20-vvIBDV-VP2.

The coding region of the vvIBDV VP2 gene was inserted into the rHN20 genome to assess whether rHN20 could deliver a protective antigen after adding a CMV promoter and poly-A to rescue the recombinant virus rHN20-vvIBDV-VP2 (Figure 6A). Using PCR and IFA, we found that the vvIBDV VP2 expression cassette was precisely inserted at the natural 1,966-bp deletion, and the vvIBDV VP2 protein could be expressed (Figures 6B,C). The growth kinetics of rHN20-vvIBDV-VP2 were delayed, and the virus titer was approximately a 1-log defect compared with rHN20 (Figure 6D).

### Protective Efficacy of Live rHN20-vvIBDV-VP2 Against FAdV-4 and vvIBDV

**FIGURE 7:**
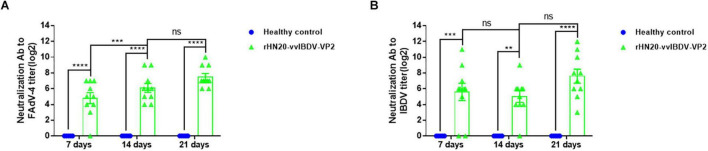
Neutralizing antibody titers of live rHN20-vvIBDV-VP2. Neutralizing antibody titers to **(A)** FAdV-4 and **(B)** IBDV in two-week-old SPF chickens intramuscularly injected with live rHN20-vvIBDV-VP. **, ***, ****, significance at *P* < 0.01, *P* < 0.001, and *P* < 0.0001, respectively; ns, no significance.

**FIGURE 8:**
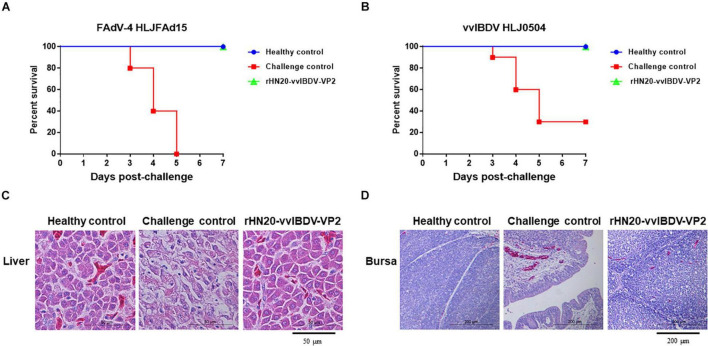
Efficacy of live rHN20-vvIBDV-VP2 vaccine against FAdV-4 and vvIBDV. Immunized chickens were challenged with FAdV-4 or IBDV. Survival rates of chickens after **(A)** FAdV-4 or **(B)** IBDV challenge. **(C)** Pathological sections of liver from FAdV-4 challenged chickens. Scale bar, 50 μm. **(D)** Pathological sections of bursas from IBDV challenged chickens. Scale bar, 200 μm.

Chickens were vaccinated with rHN20-vvIBDV-VP2 to evaluate the protective efficacy of the rHN20-vvIBDV-VP2 recombinant virus, and serum neutralizing antibodies against FAdV-4 and vvIBDV were continuously detected. Next, they were challenged with FAdV-4 HLJFAd15 or vvIBDV HLJ0504 at 21 days post-vaccination. The virus neutralization assay showed that the immunized chicken serum had strong neutralizing activity against FAdV-4 (Figure 7A) and vvIBDV (Figure 7B). All immunized chickens did not show any clinical signs or mortality after the challenge with FAdV-4 (Figure 8A) or vvIBDV (Figure 8B). In contrast, challenged chickens showed high mortality (100% for FAdV-4 and 70% for vvIBDV). In line with these findings, no histopathological changes were evident in the liver or bursa of immunized chickens; however, FAdV-4 challenge controls showed hepatocyte necrosis (Figure 8C), whereas vvIBDV challenge controls showed bursa cell necrosis and follicular atrophy (Figure 8D).

## Discussion

Severe HHS associated with the novel genotype FAdV-4 has emerged and widely spread in China since 2015, resulting in severe economic losses to the poultry industry. Due to the high mortality in broilers (Wei et al., 2019), layers (Mei et al., 2020), and breeders (Mirzazadeh et al., 2021), it is necessary to develop an effective vaccine and therapeutic strategy to combat the novel FAdV-4. Previous studies showed that an inactivated FAdV-4 oil-emulsified vaccine (Pan et al., 2017b) and subunit vaccines based on fiber 2 and hexon (Ruan et al., 2018; Hu et al., 2021) could provide complete protection against FAdV-4. Recombinant *Lactococcus lactis* and *Enterococcus faecalis* expressing hexon (Jia et al., 2021) and Newcastle disease virus (NDV) expressing fiber 2 (Tian et al., 2020) were also constructed and evaluated. Given the emergence, high pathogenicity, and lack of knowledge of the non-essential regions of the novel FAdV-4, limited progress has been achieved on the development of the FAdV-4 vector. Live FAdV-4 vectored bivalent or multiple vaccines could further reduce the production cost and workload.

A fiber-2-edited live attenuated vaccine candidate was previously developed, providing efficient protection against lethal challenge with FAdV-4; however, the titer of the edited strain was significantly decreased (Xie et al., 2021), limiting the application of the vaccine. In our previous studies, the hexon gene was identified as critical for FAdV-4 pathogenicity, and a non-pathogenic hexon-replaced chimeric strain rHN20 was rescued, showing similar virus titers compared with the wild-type strain (Zhang Y. et al., 2021). Nevertheless, the unknown non-essential regions for replication have limited the FAdV-4 vector development. In the present study, 10 ORFs at the left end and 13 ORFs at the right end of the novel FAdV-4 genome were separately deleted and identified as non-essential regions for virus replication based on an EGFP-indicator virus, which is the first report to systemically identify the non-essential ORFs for FAdV-4 replication, providing preliminary insertion sites for exogenous genes and essential information for gene function studies. These results were consistent with investigations on other serotypes of FAdVs, which showed that 16 of the 22 terminal ORFs (ORF1–4, ORF7–18) can be knocked out by point mutation in FAdV-1 (François et al., 2001) and that six ORFs are dispensable for non-pathogenic FAdV-9 (Corredor and Nagy, 2010).

To further improve the efficiency and capacity of foreign gene insertion, we successfully replaced seven combinations of ORFs with EGFP without affecting the immunogenicity of the vector backbone. Moreover, the VP2 gene of vvIBDV was delivered by the FAdV-4 vector; the VP2 protein was successfully expressed, and the recombinant rHN20-vvIBDV-VP2 provided complete protection against the FAdV-4 and vvIBDV challenges. Evaluation of rHN20-vvIBDV-VP2 revealed the delivery capacity of the FAdV-4 vector and the immunogenicity of its backbone. Besides, the combination of rHN20 and vvIBDV-VP2 further increased the capacity and application availability of the vector. Meanwhile, the seven combinations provided more possibilities for inserting other exogenous antigen genes to develop multivalent vaccines. The FAdV-4 vector developed in the present study protected HHS and successfully delivered exogenous genes against the related diseases.

## Conclusion

In conclusion, we systematically identified all ORFs located at both ends of the emerging novel FAdV-4 genome as non-essential genes for virus replication. To further improve the feasibility of FAdV-4 as a vaccine vector, seven combinations of ORFs were successfully replaced with EGFP without affecting the immunogenicity of the vector backbone. Finally, a recombinant rHN20-vvIBDV-VP2 strain expressing the VP2 protein of vvIBDV provided complete protection against the FAdV-4 and vvIBDV challenges, highlighting that the FAdV-4 vector could sufficiently protect against HHS and efficiently deliver the exogenous gene. Overall, these findings systemically identified the non-essential ORFs for FAdV-4 replication and seven foreign gene insertion regions, providing valuable information for an in-depth understanding of virus pathogenesis and the development of multivalent vaccines.

## Data Availability Statement

The original contributions presented in the study are included in the article/supplementary material, further inquiries can be directed to the corresponding author/s.

## Ethics Statement

The animal study was reviewed and approved by the Ethical and Animal Welfare Committee of HVRI.

## Author Contributions

XW conceived and designed the experiments. QP, YuZ, and AL performed the experiments. QP and KL analyzed the data. HC, CL, YaZ, and LG contributed reagents, materials, and analysis tools. QP wrote the manuscript. YG and XQ were involved in the interpretation of the results and critically read the manuscript. All authors contributed to the article and approved the submitted version.

## Conflict of Interest

The authors declare that the research was conducted in the absence of any commercial or financial relationships that could be construed as a potential conflict of interest.

## Publisher’s Note

All claims expressed in this article are solely those of the authors and do not necessarily represent those of their affiliated organizations, or those of the publisher, the editors and the reviewers. Any product that may be evaluated in this article, or claim that may be made by its manufacturer, is not guaranteed or endorsed by the publisher.

## References

[B1] Arnone C. M. Polito V. A. Mastronuzzi A. Carai A. Diomedi F. C. Antonucci L. (2021). Oncolytic adenovirus and gene therapy with EphA2-BiTE for the treatment of pediatric high-grade gliomas. *J. Immunother. Cancer* 9:e001930. 10.1136/jitc-2020-001930 PMC8108682 33963009

[B2] Baigent S. J. Petherbridge L. J. Howes K. Smith L. P. Currie R. J. Nair V. K. (2005). Absolute quantitation of Marek’s disease virus genome copy number in chicken feather and lymphocyte samples using real-time PCR. *J. Virol. Methods* 123 53–64. 10.1016/j.jviromet.2004.08.019 15582699

[B3] Boyle D. B. Heine H. G. (1993). Recombinant fowlpox virus vaccines for poultry. *Immunol. Cell Biol.* 71 391–397. 10.1038/icb.1993.45 8270268 PMC7165850

[B4] Corredor J. C. Nagy E. (2010). The non-essential left end region of the fowl adenovirus 9 genome is suitable for foreign gene insertion/replacement. *Virus Res.* 149 167–174. 10.1016/j.virusres.2010.01.014 20132849

[B5] Davison A. J. Benkő M. Harrach B. (2003). Genetic content and evolution of adenoviruses. *J. Gen. Virol.* 84 2895–2908. 10.1099/vir.0.19497-0 14573794

[B6] François A. Eterradossi N. Delmas B. Payet V. Langlois P. (2001). Construction of avian adenovirus CELO recombinants in cosmids. *J. Virol.* 75 5288–5301. 10.1128/jvi.75.11.5288-5301.2001 11333910 PMC114934

[B7] Gao L. Qi X. Li K. Gao H. Gao Y. Qin L. (2011). Development of a tailored vaccine against challenge with very virulent infectious bursal disease virus of chickens using reverse genetics. *Vaccine* 29 5550–5557. 10.1016/j.vaccine.2011.04.106 21658423

[B8] Greber U. F. (2020). Adenoviruses - Infection, pathogenesis and therapy. *FEBS Lett.* 594 1818–1827. 10.1002/1873-3468.13849 32538496

[B9] Herold B. C. Marcellino D. Marcelin G. Wilson P. Burrow C. Satlin L. M. (2002). Herpes simplex virus as a model vector system for gene therapy in renal disease. *Kidney Int.* 61 S3–S8. 10.1046/j.1523-1755.2002.0610s1003.x 11841605

[B10] Hu J. Li G. Xi W. Cai L. Rong M. Li H. (2021). Development of a subunit vaccine based on fiber2 and hexon against fowl adenovirus serotype 4. *Virus Res.* 305:198552. 10.1016/j.virusres.2021.198552 34454971

[B11] Jia Z. Ma C. Yang X. Pan X. Li G. Ma D. (2021). Oral immunization of recombinant Lactococcus lactis and Enterococcus faecalis expressing dendritic cell targeting peptide and Hexon protein of fowl Adenovirus 4 induces protective immunity against homologous infection. *Front. Vet. Sci.* 8:632218. 10.3389/fvets.2021.632218 33708811 PMC7940690

[B12] Lévy Y. Lacabaratz C. Ellefsen-Lavoie K. Stöhr W. Lelièvre J. D. Bart P. A. (2020). Optimal priming of poxvirus vector (NYVAC)-based HIV vaccine regimens for T cell responses requires three DNA injections. Results of the randomized multicentre EV03/ANRS VAC20 Phase I/II Trial. *PLoS Pathog.* 16:e1008522. 10.1371/journal.ppat.1008522 32589686 PMC7319597

[B13] Li J. X. Hou L. H. Meng F. Y. Wu S. P. Hu Y. M. Liang Q. (2017). Immunity duration of a recombinant adenovirus type-5 vector-based Ebola vaccine and a homologous prime-boost immunisation in healthy adults in China: final report of a randomised, double-blind, placebo-controlled, phase 1 trial. *Lancet Glob. Health* 5 e324–e334. 10.1016/s2214-109x(16)30367-9 28017642

[B14] Li K. Liu Y. Liu C. Gao L. Zhang Y. Cui H. (2016). Recombinant Marek’s disease virus type 1 provides full protection against very virulent Marek’s and infectious bursal disease viruses in chickens. *Sci. Rep.* 6:39263. 10.1038/srep39263 27982090 PMC5159867

[B15] Li L. Wang J. Chen P. Zhang S. Sun J. Yuan W. (2018). Pathogenicity and molecular characterization of a fowl adenovirus 4 isolated from chicken associated with IBH and HPS in China. *BMC Vet. Res.* 14:400. 10.1186/s12917-018-1733-4 30547794 PMC6295067

[B16] Liu Y. Wan W. Gao D. Li Y. Yang X. Liu H. (2016). Genetic characterization of novel fowl aviadenovirus 4 isolates from outbreaks of hepatitis-hydropericardium syndrome in broiler chickens in China. *Emerg. Microbes Infect.* 5:e117. 10.1038/emi.2016.115 27876783 PMC5148019

[B17] Mei C. Xian H. Blackall P. J. Hu W. Zhang X. Wang H. (2020). Concurrent infection of Avibacterium paragallinarum and fowl adenovirus in layer chickens. *Poult. Sci.* 99 6525–6532. 10.1016/j.psj.2020.09.033 33248567 PMC7704954

[B18] Michou A. I. Lehrmann H. Saltik M. Cotten M. (1999). Mutational analysis of the avian adenovirus CELO, which provides a basis for gene delivery vectors. *J. Virol.* 73 1399–1410. 10.1128/jvi.73.2.1399-1410.1999 9882345 PMC103964

[B19] Mirzazadeh A. Grafl B. Berger E. Schachner A. Hess M. (2021). Longitudinal serological monitoring of commercial broiler breeders for fowl adenoviruses (FAdVs)-presence of antibodies is linked with virus excretion. *Avian Dis.* 65 177–187. 10.1637/aviandiseases-D-20-00107 34339138

[B20] Niu Y. J. Sun W. Zhang G. H. Qu Y. J. Wang P. F. Sun H. L. (2016). Hydropericardium syndrome outbreak caused by fowl adenovirus serotype 4 in China in 2015. *J. Gen. Virol.* 97 2684–2690. 10.1099/jgv.0.000567 27473862

[B21] Pan Q. Liu L. Gao Y. Liu C. Qi X. Zhang Y. (2017a). Characterization of a hypervirulent fowl adenovirus 4 with the novel genotype newly prevalent in China and establishment of reproduction infection model of hydropericardium syndrome in chickens. *Poult. Sci.* 96 1581–1588. 10.3382/ps/pew431 28339951

[B22] Pan Q. Yang Y. Gao Y. Qi X. Liu C. Zhang Y. (2017b). An inactivated novel genotype fowl adenovirus 4 protects chickens against the hydropericardium syndrome that recently emerged in China. *Viruses* 9:216. 10.3390/v9080216 PMC5580473 28786949

[B23] Pan Q. Yang Y. Shi Z. Liu L. Gao Y. Qi X. (2017c). Different dynamic distribution in chickens and ducks of the hypervirulent, novel genotype fowl adenovirus serotype 4 recently emerged in China. *Front. Microbiol.* 8:1005. 10.3389/fmicb.2017.01005 28634474 PMC5459905

[B24] Pan Q. Wang J. Gao Y. Cui H. Liu C. Qi X. (2018). The Natural Large Genomic Deletion Is Unrelated to the Increased Virulence of the Novel Genotype Fowl Adenovirus 4 Recently Emerged in China. *Viruses* 10:494. 10.3390/v10090494 PMC6165077 30217040

[B25] Ruan S. Zhao J. Yin X. He Z. Zhang G. (2018). A subunit vaccine based on fiber-2 protein provides full protection against fowl adenovirus serotype 4 and induces quicker and stronger immune responses than an inactivated oil-emulsion vaccine. *Infect. Genet. Evol.* 61 145–150. 10.1016/j.meegid.2018.03.031 29614324

[B26] Schachner A. Matos M. Grafl B. Hess M. (2018). Fowl adenovirus-induced diseases and strategies for their control - a review on the current global situation. *Avian Pathol.* 47 111–126. 10.1080/03079457.2017.1385724 28950714

[B27] Steer P. A. Kirkpatrick N. C. O’Rourke D. Noormohammadi A. H. (2009). Classification of fowl adenovirus serotypes by use of high-resolution melting-curve analysis of the hexon gene region. *J. Clin. Microbiol.* 47 311–321. 10.1128/jcm.01567-08 19036935 PMC2643661

[B28] Sun W. Leist S. R. McCroskery S. Liu Y. Slamanig S. Oliva J. (2020). Newcastle disease virus (NDV) expressing the spike protein of SARS-CoV-2 as a live virus vaccine candidate. *EBioMedicine* 62:103132. 10.1016/j.ebiom.2020.103132 33232870 PMC7679520

[B29] Tatsis N. Ertl H. C. (2004). Adenoviruses as vaccine vectors. *Mol. Ther.* 10 616–629. 10.1016/j.ymthe.2004.07.013 15451446 PMC7106330

[B30] Taylor J. Weinberg R. Languet B. Desmettre P. Paoletti E. (1988). Recombinant fowlpox virus inducing protective immunity in non-avian species. *Vaccine* 6 497–503. 10.1016/0264-410x(88)90100-4 2854338

[B31] Tian K. Y. Guo H. F. Li N. Zhang Y. H. Wang Z. Wang B. (2020). Protection of chickens against hepatitis-hydropericardium syndrome and Newcastle disease with a recombinant Newcastle disease virus vaccine expressing the fowl adenovirus serotype 4 fiber-2 protein. *Vaccine* 38 1989–1997. 10.1016/j.vaccine.2020.01.006 31948818

[B32] Tostanoski L. H. Gralinski L. E. Martinez D. R. Schaefer A. Mahrokhian S. H. Li Z. (2021). Protective efficacy of rhesus adenovirus COVID-19 vaccines against mouse-adapted SARS-CoV-2. *J. Virol.* [Epub Online ahead of print]. 10.1128/jvi.00974-21 34523968 PMC8577371

[B33] Wang J. Zaheer I. Saleemi M. K. Qi X. Gao Y. Cui H. (2020). The first complete genome sequence and pathogenicity characterization of fowl adenovirus 11 from chickens with inclusion body hepatitis in Pakistan. *Vet. Microbiol.* 244:108670. 10.1016/j.vetmic.2020.108670 32402334

[B34] Wei Z. Liu H. Diao Y. Li X. Zhang S. Gao B. (2019). Pathogenicity of fowl adenovirus (FAdV) serotype 4 strain SDJN in Taizhou geese. *Avian Pathol.* 48 477–485. 10.1080/03079457.2019.1625305 31155930

[B35] Wu S. Huang J. Zhang Z. Wu J. Zhang J. Hu H. (2021). Safety, tolerability, and immunogenicity of an aerosolised adenovirus type-5 vector-based COVID-19 vaccine (Ad5-nCoV) in adults: preliminary report of an open-label and randomised phase 1 clinical trial. *Lancet Infect. Dis.* [Epub Online ahead of Print]. 10.1016/s1473-3099(21)00396-0 PMC8313090 34324836

[B36] Xia J. Yao K. C. Liu Y. Y. You G. J. Li S. Y. Liu P. (2017). Isolation and molecular characterization of prevalent Fowl adenovirus strains in southwestern China during 2015-2016 for the development of a control strategy. *Emerg. Microbes Infect.* 6:e103. 10.1038/emi.2017.91 29184155 PMC5717092

[B37] Xie Q. Cao S. Zhang W. Wang W. Li L. Kan Q. (2021). A novel fiber-2-edited live attenuated vaccine candidate against the highly pathogenic serotype 4 fowl adenovirus. *Vet. Res.* 52:35. 10.1186/s13567-021-00907-z 33640033 PMC7912893

[B38] Zhang X. Bo Z. Meng C. Chen Y. Zhang C. Cao Y. (2021). Generation and evaluation of recombinant Thermostable Newcastle Disease Virus Expressing the HA of H9N2 Avian Influenza Virus. *Viruses* 13:1606. 10.3390/v13081606 PMC8402907 34452473

[B39] Zhang Y. Liu A. Wang Y. Cui H. Gao Y. Qi X. (2021). A single amino acid at residue 188 of the hexon protein is responsible for the pathogenicity of the emerging novel virus fowl adenovirus 4. *J. Virol.* 95:e0060321. 10.1128/jvi.00603-21 34133902 PMC8354325

